# Poly[aqua[μ_2_-1,2-bis­(imidazol-1-yl­methyl)benzene-κ^2^ *N* ^3^:*N* ^3′^](μ_2_-5-bromo­benzene-1,3-dicarboxyl­ato-κ^3^ *O* ^1^,*O* ^1′^:*O* ^3^)nickel(II)]

**DOI:** 10.1107/S1600536809016286

**Published:** 2009-05-07

**Authors:** Kun Zhu, Hong Chen, Guang-Xiang Liu

**Affiliations:** a Anhui Key Laboratory of Functional Coordination Compounds, School of Chemistry and Chemical Engineering, Anqing Normal University, Anqing 246003, People’s Republic of China

## Abstract

In the two-dimensional title coordination polymer, [Ni(C_8_H_3_BrO_4_)(C_14_H_14_N_4_)(H_2_O)]_*n*_, the Ni^II^ atom adopts a distorted octa­hedral geometry, being ligated by three O atoms from two different 5-bromo­benzene-1,3-dicarboxyl­ate ligands, two N atoms from two 1,2-bis­(imidazol-1-ylmeth­yl)benzene ligands and one coordinated water mol­ecule. The Ni atoms are bridged by the 5-bromo­benzene-1,3-dicarboxyl­ate ligands, forming chains, which are further linked by 1,2-bis­(imidazol-1-ylmeth­yl)benzene, generating a layer structure parallel to (001).

## Related literature

For general background to self-assembly coordination polymers with metal ions and bis­(imidazole) ligands inter­connected by flexible spacers, see: Qi *et al.* (2008 ▶); Liu *et al.* (2009 ▶). For the role played by different organic anions in directing the final structure and topology, see: Hu *et al.* (2008 ▶). For related structures, see: Liu *et al.* (2008 ▶).
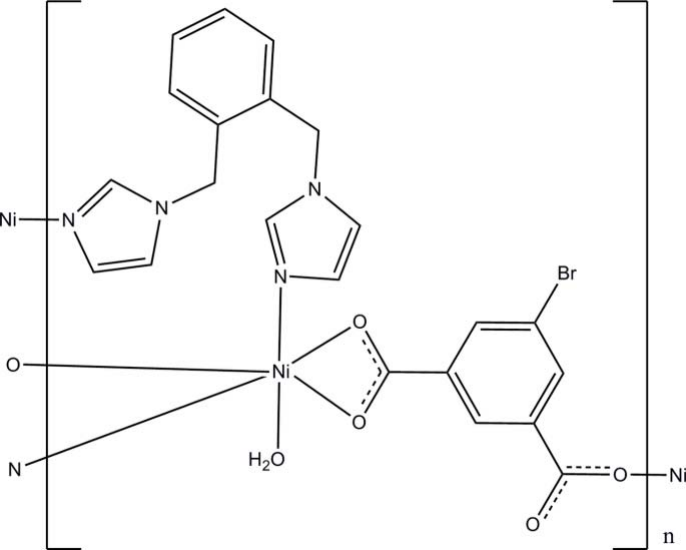


## Experimental

### 

#### Crystal data

[Ni(C_8_H_3_BrO_4_)(C_14_H_14_N_4_)(H_2_O)]
*M* *_r_* = 558.03
Triclinic, 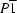

*a* = 9.1374 (12) Å
*b* = 10.1394 (14) Å
*c* = 12.9642 (18) Å
α = 80.046 (2)°
β = 83.233 (2)°
γ = 70.004 (2)°
*V* = 1109.5 (3) Å^3^
*Z* = 2
Mo *K*α radiation
μ = 2.72 mm^−1^
*T* = 293 K
0.26 × 0.20 × 0.18 mm

#### Data collection

Bruker SMART APEX CCD area-detector diffractometer
Absorption correction: multi-scan (*SADABS*: Bruker, 1997 ▶) *T*_min_ = 0.538, *T*_max_ = 0.641
8172 measured reflections
4055 independent reflections
3029 reflections with *I* > 2σ(*I*)
*R*_int_ = 0.029

#### Refinement

*R*[*F* ^2^ > 2σ(*F* ^2^)] = 0.040
*wR*(*F* ^2^) = 0.085
*S* = 1.04
4055 reflections
291 parameters
H atoms treated by a mixture of independent and constrained refinement
Δρ_max_ = 0.64 e Å^−3^
Δρ_min_ = −1.12 e Å^−3^

### 

Data collection: *SMART* (Bruker, 1997 ▶); cell refinement: *SAINT* (Bruker, 1997 ▶); data reduction: *SAINT*; program(s) used to solve structure: *SHELXS97* (Sheldrick, 2008 ▶); program(s) used to refine structure: *SHELXL97* (Sheldrick, 2008 ▶); molecular graphics: *SHELXTL* (Sheldrick, 2008 ▶); software used to prepare material for publication: *SHELXTL*.

## Supplementary Material

Crystal structure: contains datablocks I, global. DOI: 10.1107/S1600536809016286/at2774sup1.cif

Structure factors: contains datablocks I. DOI: 10.1107/S1600536809016286/at2774Isup2.hkl

Additional supplementary materials:  crystallographic information; 3D view; checkCIF report

## Figures and Tables

**Fig. 1.**
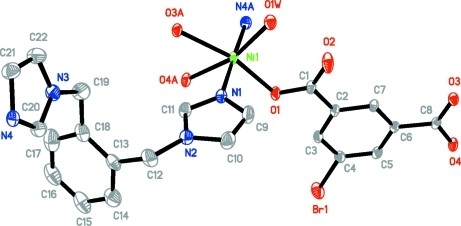
The asymmetric unit of the title compound, extended to show the Ni coordination. Displacement ellipsoids are drawn at the 30% probability level. H atoms have been omitted for clarity. [Symmetry code: (i) x - 1, y + 1, z; (ii) x, y - 1, z.]

**Fig. 2.**
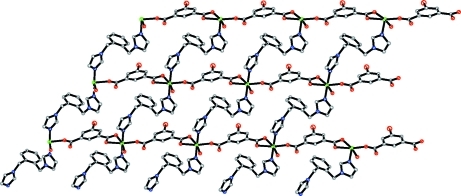
The two-dimensional layer structure of the title compound.

## supplementary crystallographic information

### Comment

The self-assembly of coordination polymers has attracted considerable attention in the past decade. This arises mainly for their various intriguing topological structures and their potential applications in material chemistry. Recently significant work has been carried out by using metal ions assembly with bis(imidazole) ligands interconnected by flexible spacers (Qi *et al.*, 2008; Liu *et al.*, 2009). From careful inspection of the reported cases, we found that: the ligand exhibits a special ability to formulate the compounds, and different organic anions play an important role in directing the final structures and topologies (Hu *et al.*, 2008). Inspired by the aftermentioned considerations, 1,2-bis(imidazol-1-ylmethyl)benzene was chosen as neutral ligands, 5-bromobenzene-1,3-dicarboxylate were chosen as co-ligands to construct the title complex (I).

The title coordination polymer is a two-dimensional layer coordination polymer. The Ni^II^ atom adopts a distorted octahedral geometry, being ligated by three O atoms from two different 5-bromobenzene-1,3-dicarboxylate ligand, two N atoms from two 1,2-bis(imidazol-1-ylmethyl)benzene and one coordinated water molecule, as shown in Fig. 1. The Ni atoms are bridged by 5-bromobenzene-1,3-dicarboxylate ligand to form one-dimensional chain, which are further linked by 1,2-bis(imidazol-1-ylmethyl)benzene to generate a two-dimensional layer structure, as shown in Fig. 2.

### Experimental

A mixture of Ni(NO_3_)_2._6H_2_O (58.2 mg, 0.2 mmol), 5-bromobenzene-1,3-dicarboxylate acid (33.0 mg, 0.1 mmol), 1,2-bis(imidazol-1-ylmethyl)benzene (23.8 mg, 0.1 mmol), NaOH (8 mg, 0.2 mmol) and H_2_O (15 ml) was added in a Teflon-lined stainless steel vessel. The vessel was sealed and heated for 3 d at 433 K. After the mixture was slowly cooled to room temperature, green block crystals were obtained in the yield of *ca* 67% based on Ni.

### Refinement

H atoms were positioned geometrically, with C—H = 0.93 and 0.96 Å for aromatic and methyl H atoms, respectively, and constrained to ride on their parent atoms, with *U*_iso_(H) = xU_eq_(C), where *x* = 1.5 for methyl H and *x* = 1.2 for aromatic H atoms. The deepest hole is located 1.12 Å from atom C16.

### Crystal data

**Table:**

[Ni(C_8_H_3_BrO_4_)(C_14_H_14_N_4_)(H_2_O)]	*Z* = 2
*M**_r_* = 558.03	*F*(000) = 564
Triclinic, *P*1	*D*_x_ = 1.670 Mg m^−^^3^
Hall symbol: -P 1	Mo *K*α radiation, λ = 0.71073 Å
*a* = 9.1374 (12) Å	Cell parameters from 2114 reflections
*b* = 10.1394 (14) Å	θ = 2.4–24.0°
*c* = 12.9642 (18) Å	µ = 2.72 mm^−^^1^
α = 80.046 (2)°	*T* = 293 K
β = 83.233 (2)°	Block, green
γ = 70.004 (2)°	0.26 × 0.20 × 0.18 mm
*V* = 1109.5 (3) Å^3^	

### Data collection

**Table:**

Bruker SMART APEX CCD area-detector diffractometer	4055 independent reflections
Radiation source: sealed tube	3029 reflections with *I* > 2σ(*I*)
graphite	*R*_int_ = 0.029
φ and ω scans	θ_max_ = 25.5°, θ_min_ = 1.6°
Absorption correction: multi-scan (*SADABS*: Bruker, 1997)	*h* = −11→9
*T*_min_ = 0.538, *T*_max_ = 0.641	*k* = −12→11
8172 measured reflections	*l* = −15→15

### Refinement

**Table:**

Refinement on *F*^2^	Primary atom site location: structure-invariant direct methods
Least-squares matrix: full	Secondary atom site location: difference Fourier map
*R*[*F*^2^ > 2σ(*F*^2^)] = 0.040	Hydrogen site location: inferred from neighbouring sites
*wR*(*F*^2^) = 0.085	H atoms treated by a mixture of independent and constrained refinement
*S* = 1.04	*w* = 1/[σ^2^(*F*_o_^2^) + (0.029*P*)^2^ + 0.735*P*] where *P* = (*F*_o_^2^ + 2*F*_c_^2^)/3
4055 reflections	(Δ/σ)_max_ < 0.001
291 parameters	Δρ_max_ = 0.64 e Å^−^^3^
0 restraints	Δρ_min_ = −1.12 e Å^−^^3^

### Special details

**Table:**

Geometry. All e.s.d.'s (except the e.s.d. in the dihedral angle between two l.s. planes) are estimated using the full covariance matrix. The cell e.s.d.'s are taken into account individually in the estimation of e.s.d.'s in distances, angles and torsion angles; correlations between e.s.d.'s in cell parameters are only used when they are defined by crystal symmetry. An approximate (isotropic) treatment of cell e.s.d.'s is used for estimating e.s.d.'s involving l.s. planes.
Refinement. Refinement of *F*^2^ against ALL reflections. The weighted *R*-factor *wR* and goodness of fit *S* are based on *F*^2^, conventional *R*-factors *R* are based on *F*, with *F* set to zero for negative *F*^2^. The threshold expression of *F*^2^ > σ(*F*^2^) is used only for calculating *R*-factors(gt) *etc*. and is not relevant to the choice of reflections for refinement. *R*-factors based on *F*^2^ are statistically about twice as large as those based on *F*, and *R*- factors based on ALL data will be even larger.

### Fractional atomic coordinates and isotropic or equivalent isotropic displacement parameters (Å^2^)

**Table:**

	*x*	*y*	*z*	*U*_iso_*/*U*_eq_	
Ni1	−0.11002 (5)	0.18147 (4)	0.34656 (3)	0.02475 (13)	
N1	0.1280 (3)	0.0814 (3)	0.3229 (2)	0.0286 (7)	
N2	0.3530 (3)	−0.0730 (3)	0.2776 (2)	0.0332 (7)	
N3	0.4261 (3)	−0.5489 (3)	0.3554 (2)	0.0372 (8)	
N4	0.6546 (3)	−0.7159 (3)	0.3708 (2)	0.0313 (7)	
O1	−0.0886 (3)	0.3683 (2)	0.27370 (18)	0.0326 (6)	
O2	−0.0881 (4)	0.4666 (3)	0.4146 (2)	0.0581 (9)	
O3	−0.1442 (3)	0.9777 (2)	0.37521 (17)	0.0287 (5)	
O4	−0.1529 (3)	1.0986 (2)	0.21699 (17)	0.0308 (6)	
O1W	−0.0840 (3)	0.2116 (3)	0.4966 (2)	0.0341 (7)	
Br1	−0.14928 (6)	0.76345 (5)	−0.06669 (3)	0.06060 (18)	
C1	−0.0961 (4)	0.4705 (3)	0.3195 (3)	0.0282 (8)	
C2	−0.1196 (4)	0.6114 (3)	0.2502 (3)	0.0251 (8)	
C3	−0.1282 (4)	0.6229 (4)	0.1422 (3)	0.0291 (8)	
H3	−0.1227	0.5444	0.1120	0.035*	
C4	−0.1448 (4)	0.7516 (4)	0.0806 (3)	0.0306 (8)	
C5	−0.1547 (4)	0.8709 (4)	0.1226 (3)	0.0309 (8)	
H5	−0.1661	0.9569	0.0796	0.037*	
C6	−0.1473 (4)	0.8599 (3)	0.2307 (3)	0.0239 (7)	
C7	−0.1303 (4)	0.7308 (3)	0.2944 (3)	0.0242 (7)	
H7	−0.1260	0.7240	0.3665	0.029*	
C8	−0.1508 (4)	0.9869 (3)	0.2772 (3)	0.0247 (8)	
C9	0.2459 (5)	0.1249 (4)	0.3411 (3)	0.0454 (11)	
H9	0.2329	0.2076	0.3682	0.055*	
C10	0.3845 (5)	0.0317 (4)	0.3144 (3)	0.0489 (11)	
H10	0.4826	0.0376	0.3199	0.059*	
C11	0.1973 (4)	−0.0380 (4)	0.2842 (3)	0.0342 (9)	
H11	0.1444	−0.0919	0.2638	0.041*	
C12	0.4673 (4)	−0.2047 (4)	0.2455 (3)	0.0468 (11)	
H12A	0.4975	−0.2743	0.3074	0.056*	
H12B	0.5601	−0.1848	0.2128	0.056*	
C13	0.4039 (4)	−0.2660 (4)	0.1699 (3)	0.0388 (10)	
C14	0.4232 (5)	−0.2198 (5)	0.0633 (4)	0.0578 (12)	
H14	0.4746	−0.1540	0.0412	0.069*	
C15	0.3664 (5)	−0.2713 (5)	−0.0108 (4)	0.060	
H15	0.3765	−0.2380	−0.0819	0.072*	
C16	0.2960 (4)	−0.3706 (3)	0.0223 (3)	0.060	
H16	0.2594	−0.4069	−0.0267	0.072*	
C17	0.2782 (4)	−0.4182 (3)	0.1269 (3)	0.0678 (15)	
H17	0.2305	−0.4871	0.1477	0.081*	
C18	0.3299 (4)	−0.3658 (4)	0.2031 (3)	0.0427 (10)	
C19	0.3027 (5)	−0.4184 (4)	0.3173 (4)	0.0550 (12)	
H19A	0.2961	−0.3452	0.3587	0.066*	
H19B	0.2035	−0.4356	0.3275	0.066*	
C20	0.5718 (4)	−0.6006 (4)	0.3131 (3)	0.0400 (10)	
H20	0.6095	−0.5598	0.2505	0.048*	
C21	0.5557 (5)	−0.7398 (4)	0.4541 (3)	0.0456 (10)	
H21	0.5813	−0.8154	0.5082	0.055*	
C22	0.4150 (5)	−0.6367 (5)	0.4458 (3)	0.0491 (11)	
H22	0.3280	−0.6278	0.4926	0.059*	
H1WA	−0.067 (5)	0.285 (5)	0.483 (3)	0.059 (16)*	
H1WB	−0.008 (5)	0.158 (4)	0.529 (3)	0.047 (14)*	

### Atomic displacement parameters (Å^2^)

**Table:**

	*U*^11^	*U*^22^	*U*^33^	*U*^12^	*U*^13^	*U*^23^
Ni1	0.0333 (3)	0.0136 (2)	0.0266 (2)	−0.00590 (19)	−0.00584 (19)	−0.00212 (18)
N1	0.0322 (18)	0.0215 (16)	0.0319 (16)	−0.0074 (14)	−0.0049 (13)	−0.0040 (13)
N2	0.0253 (18)	0.0315 (18)	0.0413 (18)	−0.0055 (14)	−0.0041 (14)	−0.0079 (14)
N3	0.0277 (18)	0.0291 (18)	0.048 (2)	−0.0014 (14)	−0.0009 (15)	−0.0063 (15)
N4	0.0332 (18)	0.0224 (17)	0.0362 (17)	−0.0061 (14)	−0.0031 (14)	−0.0044 (14)
O1	0.0496 (16)	0.0163 (12)	0.0325 (13)	−0.0115 (12)	−0.0013 (12)	−0.0046 (10)
O2	0.118 (3)	0.0240 (15)	0.0365 (16)	−0.0248 (16)	−0.0304 (17)	0.0041 (12)
O3	0.0397 (15)	0.0172 (12)	0.0281 (13)	−0.0078 (11)	−0.0057 (11)	−0.0015 (10)
O4	0.0452 (16)	0.0179 (13)	0.0313 (13)	−0.0116 (12)	−0.0099 (11)	−0.0011 (10)
O1W	0.0465 (19)	0.0231 (16)	0.0311 (15)	−0.0087 (15)	−0.0100 (13)	−0.0006 (12)
Br1	0.1137 (5)	0.0440 (3)	0.0264 (2)	−0.0292 (3)	−0.0036 (2)	−0.00487 (19)
C1	0.035 (2)	0.0182 (19)	0.032 (2)	−0.0078 (16)	−0.0067 (16)	−0.0026 (15)
C2	0.0250 (19)	0.0179 (18)	0.0340 (19)	−0.0079 (15)	−0.0046 (15)	−0.0045 (15)
C3	0.036 (2)	0.0230 (19)	0.0308 (19)	−0.0107 (16)	−0.0016 (16)	−0.0076 (15)
C4	0.041 (2)	0.029 (2)	0.0225 (18)	−0.0123 (17)	−0.0039 (16)	−0.0030 (15)
C5	0.043 (2)	0.0176 (18)	0.0308 (19)	−0.0116 (17)	−0.0045 (17)	0.0044 (15)
C6	0.0235 (19)	0.0167 (17)	0.0319 (19)	−0.0069 (15)	−0.0033 (15)	−0.0031 (14)
C7	0.0260 (19)	0.0187 (18)	0.0283 (18)	−0.0070 (15)	−0.0077 (15)	−0.0016 (14)
C8	0.0231 (19)	0.0146 (18)	0.035 (2)	−0.0040 (14)	−0.0050 (15)	−0.0012 (15)
C9	0.047 (3)	0.037 (2)	0.062 (3)	−0.018 (2)	−0.005 (2)	−0.018 (2)
C10	0.038 (3)	0.050 (3)	0.067 (3)	−0.022 (2)	−0.004 (2)	−0.016 (2)
C11	0.030 (2)	0.029 (2)	0.046 (2)	−0.0084 (17)	−0.0084 (17)	−0.0067 (17)
C12	0.026 (2)	0.046 (3)	0.063 (3)	0.0006 (19)	−0.003 (2)	−0.018 (2)
C13	0.031 (2)	0.035 (2)	0.041 (2)	0.0059 (18)	−0.0073 (18)	−0.0122 (19)
C14	0.063 (3)	0.037 (3)	0.056 (3)	0.003 (2)	−0.004 (2)	−0.001 (2)
C15	0.059	0.050	0.054	0.015	−0.024	−0.016
C16	0.059	0.050	0.054	0.015	−0.024	−0.016
C17	0.038 (3)	0.050 (3)	0.115 (5)	−0.002 (2)	−0.015 (3)	−0.029 (3)
C18	0.025 (2)	0.034 (2)	0.061 (3)	0.0076 (18)	−0.0108 (19)	−0.013 (2)
C19	0.032 (2)	0.039 (3)	0.079 (3)	0.003 (2)	0.007 (2)	−0.003 (2)
C20	0.035 (2)	0.032 (2)	0.045 (2)	−0.0026 (19)	0.0000 (19)	−0.0001 (19)
C21	0.049 (3)	0.044 (3)	0.040 (2)	−0.015 (2)	−0.002 (2)	0.0038 (19)
C22	0.044 (3)	0.054 (3)	0.046 (3)	−0.016 (2)	0.005 (2)	−0.005 (2)

### Geometric parameters (Å, °)

**Table:**

Ni1—O1	2.027 (2)	C4—C5	1.381 (5)
Ni1—N4^i^	2.057 (3)	C5—C6	1.395 (5)
Ni1—N1	2.072 (3)	C5—H5	0.9300
Ni1—O1W	2.073 (3)	C6—C7	1.392 (4)
Ni1—O4^ii^	2.133 (2)	C6—C8	1.503 (4)
Ni1—O3^ii^	2.157 (2)	C7—H7	0.9300
Ni1—C8^ii^	2.456 (3)	C8—Ni1^iv^	2.456 (3)
N1—C11	1.315 (4)	C9—C10	1.345 (5)
N1—C9	1.354 (4)	C9—H9	0.9300
N2—C11	1.339 (4)	C10—H10	0.9300
N2—C10	1.361 (5)	C11—H11	0.9300
N2—C12	1.478 (4)	C12—C13	1.508 (5)
N3—C20	1.341 (4)	C12—H12A	0.9700
N3—C22	1.360 (5)	C12—H12B	0.9700
N3—C19	1.466 (5)	C13—C18	1.383 (6)
N4—C20	1.314 (4)	C13—C14	1.392 (6)
N4—C21	1.368 (5)	C14—C15	1.393 (6)
N4—Ni1^iii^	2.057 (3)	C14—H14	0.9300
O1—C1	1.260 (4)	C15—C16	1.357 (6)
O2—C1	1.237 (4)	C15—H15	0.9300
O3—C8	1.264 (4)	C16—C17	1.368 (6)
O3—Ni1^iv^	2.157 (2)	C16—H16	0.9300
O4—C8	1.255 (4)	C17—C18	1.395 (5)
O4—Ni1^iv^	2.133 (2)	C17—H17	0.9300
O1W—H1WA	0.80 (5)	C18—C19	1.507 (6)
O1W—H1WB	0.82 (4)	C19—H19A	0.9700
Br1—C4	1.897 (3)	C19—H19B	0.9700
C1—C2	1.512 (5)	C20—H20	0.9300
C2—C3	1.394 (4)	C21—C22	1.353 (5)
C2—C7	1.396 (4)	C21—H21	0.9300
C3—C4	1.377 (5)	C22—H22	0.9300
C3—H3	0.9300		
			
O1—Ni1—N4^i^	88.51 (11)	C6—C7—C2	120.1 (3)
O1—Ni1—N1	90.67 (10)	C6—C7—H7	119.9
N4^i^—Ni1—N1	178.90 (11)	C2—C7—H7	119.9
O1—Ni1—O1W	95.91 (11)	O4—C8—O3	121.2 (3)
N4^i^—Ni1—O1W	87.85 (12)	O4—C8—C6	118.9 (3)
N1—Ni1—O1W	91.50 (11)	O3—C8—C6	119.8 (3)
O1—Ni1—O4^ii^	100.66 (9)	O4—C8—Ni1^iv^	60.26 (16)
N4^i^—Ni1—O4^ii^	90.21 (10)	O3—C8—Ni1^iv^	61.33 (16)
N1—Ni1—O4^ii^	90.66 (10)	C6—C8—Ni1^iv^	170.6 (2)
O1W—Ni1—O4^ii^	163.26 (11)	C10—C9—N1	110.4 (3)
O1—Ni1—O3^ii^	162.21 (9)	C10—C9—H9	124.8
N4^i^—Ni1—O3^ii^	91.35 (10)	N1—C9—H9	124.8
N1—Ni1—O3^ii^	89.66 (10)	C9—C10—N2	106.4 (3)
O1W—Ni1—O3^ii^	101.87 (10)	C9—C10—H10	126.8
O4^ii^—Ni1—O3^ii^	61.55 (9)	N2—C10—H10	126.8
O1—Ni1—C8^ii^	131.29 (10)	N1—C11—N2	112.2 (3)
N4^i^—Ni1—C8^ii^	93.07 (11)	N1—C11—H11	123.9
N1—Ni1—C8^ii^	88.03 (11)	N2—C11—H11	123.9
O1W—Ni1—C8^ii^	132.80 (12)	N2—C12—C13	112.5 (3)
O4^ii^—Ni1—C8^ii^	30.73 (9)	N2—C12—H12A	109.1
O3^ii^—Ni1—C8^ii^	30.95 (9)	C13—C12—H12A	109.1
C11—N1—C9	104.8 (3)	N2—C12—H12B	109.1
C11—N1—Ni1	127.0 (2)	C13—C12—H12B	109.1
C9—N1—Ni1	128.2 (2)	H12A—C12—H12B	107.8
C11—N2—C10	106.2 (3)	C18—C13—C14	120.0 (4)
C11—N2—C12	126.8 (3)	C18—C13—C12	122.4 (4)
C10—N2—C12	126.9 (3)	C14—C13—C12	117.6 (4)
C20—N3—C22	106.9 (3)	C15—C14—C13	120.7 (5)
C20—N3—C19	127.8 (3)	C15—C14—H14	119.7
C22—N3—C19	125.2 (3)	C13—C14—H14	119.7
C20—N4—C21	105.2 (3)	C16—C15—C14	119.0 (4)
C20—N4—Ni1^iii^	125.5 (3)	C16—C15—H15	120.5
C21—N4—Ni1^iii^	128.8 (3)	C14—C15—H15	120.5
C1—O1—Ni1	124.9 (2)	C15—C16—C17	120.9 (3)
C8—O3—Ni1^iv^	87.72 (19)	C15—C16—H16	119.6
C8—O4—Ni1^iv^	89.01 (19)	C17—C16—H16	119.6
Ni1—O1W—H1WA	99 (3)	C16—C17—C18	121.5 (3)
Ni1—O1W—H1WB	120 (3)	C16—C17—H17	119.3
H1WA—O1W—H1WB	104 (4)	C18—C17—H17	119.2
O2—C1—O1	126.4 (3)	C13—C18—C17	118.0 (4)
O2—C1—C2	117.8 (3)	C13—C18—C19	122.7 (4)
O1—C1—C2	115.9 (3)	C17—C18—C19	119.3 (4)
C3—C2—C7	119.6 (3)	N3—C19—C18	112.9 (3)
C3—C2—C1	120.7 (3)	N3—C19—H19A	109.0
C7—C2—C1	119.8 (3)	C18—C19—H19A	109.0
C4—C3—C2	119.3 (3)	N3—C19—H19B	109.0
C4—C3—H3	120.3	C18—C19—H19B	109.0
C2—C3—H3	120.3	H19A—C19—H19B	107.8
C3—C4—C5	122.1 (3)	N4—C20—N3	111.8 (3)
C3—C4—Br1	118.7 (2)	N4—C20—H20	124.1
C5—C4—Br1	119.3 (3)	N3—C20—H20	124.1
C4—C5—C6	118.7 (3)	C22—C21—N4	109.7 (4)
C4—C5—H5	120.7	C22—C21—H21	125.1
C6—C5—H5	120.7	N4—C21—H21	125.1
C7—C6—C5	120.2 (3)	C21—C22—N3	106.4 (4)
C7—C6—C8	120.3 (3)	C21—C22—H22	126.8
C5—C6—C8	119.5 (3)	N3—C22—H22	126.8
			
O1—Ni1—N1—C11	−131.0 (3)	C7—C6—C8—O3	−2.7 (5)
N4^i^—Ni1—N1—C11	−173 (47)	C5—C6—C8—O3	179.9 (3)
O1W—Ni1—N1—C11	133.1 (3)	C7—C6—C8—Ni1^iv^	91.6 (15)
O4^ii^—Ni1—N1—C11	−30.3 (3)	C5—C6—C8—Ni1^iv^	−85.7 (15)
O3^ii^—Ni1—N1—C11	31.2 (3)	C11—N1—C9—C10	−0.5 (5)
C8^ii^—Ni1—N1—C11	0.3 (3)	Ni1—N1—C9—C10	−179.1 (3)
O1—Ni1—N1—C9	47.3 (3)	N1—C9—C10—N2	0.4 (5)
N4^i^—Ni1—N1—C9	5(6)	C11—N2—C10—C9	−0.1 (5)
O1W—Ni1—N1—C9	−48.6 (3)	C12—N2—C10—C9	−175.4 (4)
O4^ii^—Ni1—N1—C9	148.0 (3)	C9—N1—C11—N2	0.5 (4)
O3^ii^—Ni1—N1—C9	−150.5 (3)	Ni1—N1—C11—N2	179.1 (2)
C8^ii^—Ni1—N1—C9	178.6 (3)	C10—N2—C11—N1	−0.2 (4)
N4^i^—Ni1—O1—C1	73.7 (3)	C12—N2—C11—N1	175.1 (3)
N1—Ni1—O1—C1	−105.6 (3)	C11—N2—C12—C13	29.1 (5)
O1W—Ni1—O1—C1	−14.0 (3)	C10—N2—C12—C13	−156.5 (4)
O4^ii^—Ni1—O1—C1	163.6 (3)	N2—C12—C13—C18	−91.9 (4)
O3^ii^—Ni1—O1—C1	163.4 (3)	N2—C12—C13—C14	88.7 (4)
C8^ii^—Ni1—O1—C1	166.5 (3)	C18—C13—C14—C15	1.1 (6)
Ni1—O1—C1—O2	14.5 (5)	C12—C13—C14—C15	−179.5 (3)
Ni1—O1—C1—C2	−164.5 (2)	C13—C14—C15—C16	−2.1 (6)
O2—C1—C2—C3	179.4 (3)	C14—C15—C16—C17	1.2 (5)
O1—C1—C2—C3	−1.5 (5)	C15—C16—C17—C18	0.6 (3)
O2—C1—C2—C7	0.5 (5)	C14—C13—C18—C17	0.7 (5)
O1—C1—C2—C7	179.6 (3)	C12—C13—C18—C17	−178.7 (3)
C7—C2—C3—C4	0.9 (5)	C14—C13—C18—C19	−178.9 (3)
C1—C2—C3—C4	−178.0 (3)	C12—C13—C18—C19	1.7 (5)
C2—C3—C4—C5	−0.6 (5)	C16—C17—C18—C13	−1.6 (4)
C2—C3—C4—Br1	177.9 (3)	C16—C17—C18—C19	178.0 (2)
C3—C4—C5—C6	0.1 (6)	C20—N3—C19—C18	20.2 (6)
Br1—C4—C5—C6	−178.4 (3)	C22—N3—C19—C18	−163.9 (4)
C4—C5—C6—C7	0.0 (5)	C13—C18—C19—N3	−94.0 (5)
C4—C5—C6—C8	177.3 (3)	C17—C18—C19—N3	86.4 (4)
C5—C6—C7—C2	0.4 (5)	C21—N4—C20—N3	0.6 (4)
C8—C6—C7—C2	−177.0 (3)	Ni1^iii^—N4—C20—N3	−172.4 (2)
C3—C2—C7—C6	−0.8 (5)	C22—N3—C20—N4	−0.1 (4)
C1—C2—C7—C6	178.1 (3)	C19—N3—C20—N4	176.4 (4)
Ni1^iv^—O4—C8—O3	7.4 (3)	C20—N4—C21—C22	−0.9 (4)
Ni1^iv^—O4—C8—C6	−169.3 (3)	Ni1^iii^—N4—C21—C22	171.8 (3)
Ni1^iv^—O3—C8—O4	−7.4 (3)	N4—C21—C22—N3	0.8 (5)
Ni1^iv^—O3—C8—C6	169.3 (3)	C20—N3—C22—C21	−0.5 (4)
C7—C6—C8—O4	174.1 (3)	C19—N3—C22—C21	−177.1 (4)
C5—C6—C8—O4	−3.3 (5)		

Symmetry codes: (i) *x*−1, *y*+1, *z*; (ii) *x*, *y*−1, *z*; (iii) *x*+1, *y*−1, *z*; (iv) *x*, *y*+1, *z*.

## References

[bb1] Bruker (1997). *SMART*, *SAINT* and *SADABS* Bruker AXS Inc., Madison, Wisconsin, USA.

[bb2] Hu, T.-L., Zou, R.-Q., Li, J.-R. & Bu, X.-H. (2008). *Dalton Trans* pp. 1302–1311. 10.1039/b716398c 18305842

[bb3] Liu, G.-X., Huang, R.-Y., Xu, H., Kong, X.-J., Huang, L.-F., Zhu, K. & Ren, X.-M. (2008). *Polyhedron*, **27**, 2327–2336.

[bb4] Liu, G.-X., Zhu, K., Chen, H., Huang, R.-Y., Xu, H. & Ren, X.-M. (2009). *Inorg. Chim. Acta*, **362**, 1605–1610.

[bb5] Qi, Y., Chi, Y. X. & Zheng, J. M. (2008). *Cryst. Growth Des* **8**, 606–611.

[bb6] Sheldrick, G. M. (2008). *Acta Cryst.* A**64**, 112–122. 10.1107/S0108767307043930 18156677

